# A Case of Acute Liver Failure Due to Artemisinin-Derived Herbal Supplements

**DOI:** 10.7759/cureus.36582

**Published:** 2023-03-23

**Authors:** Maria Jamil, Abdus Salam, Belinda Joseph Benher, Nour Nasiri, Ammad J Chaudhary

**Affiliations:** 1 Internal Medicine, Henry Ford Health System, Detroit, USA; 2 General Surgery, Aga Khan University, Peshawar, PAK; 3 Internal Medicine, Wayne State University, Detroit, USA

**Keywords:** weight loss supplement, chinese herbal supplements, transaminitis, acute liver failure, artemisinin

## Abstract

A 49-year-old female presented with malaise, nausea, vomiting, and discolored urine. She was found to have an acute liver failure with labs significant for aspartate aminotransferase (AST) of 2164, alanine aminotransferase (ALT) of 2425, alkaline phosphatase (ALP) of 106, total bilirubin of 3.6, and lactate dehydrogenase (LDH) of 2269. The international normalized ratio (INR) was also elevated at 1.9. All workup for acute liver failure was negative and it was found that she had started taking a new supplement called “Gut Health”, which contained artemisinin, for weight loss and menopausal symptoms. After discontinuing the supplements and symptomatically treating her for acute liver failure, her transaminitis resolved.

## Introduction

Artemisinin is an ancient Chinese herb with anti-malarial properties [1]. However, studies suggest it can cause liver injury, and it has been associated with elevated serum aminotransferase levels [1]. There have been reports of sudden liver injury in patients taking artemisinin derivatives [2]. We present a case of a 49-year-old female patient who developed an acute liver injury from herbal supplements containing artemisinin for weight loss and menopausal symptoms.

## Case presentation

A 49-year-old female with a past medical history of factor V mutation and deep vein thrombosis (DVT) presented to the emergency department (ED) with malaise, nausea, vomiting, and discolored urine. She had developed transaminitis a year prior to the presentation, suspected to be caused by a herbal supplement prescribed by her Naturopathic physician, which she had then stopped. Two months prior, she had started taking new supplements (“Gut Health” containing artemisinin) for weight loss and menopausal symptoms. She stated that she had taken around two capsules a day in the past two months. She had no family history of any liver or autoimmune diseases and was not taking any other supplements or medications. As shown in Table 1, her labs showed an alanine aminotransferase (ALT) of 2425, aspartate aminotransferase (AST) of 2164, total bilirubin of 3.6, alkaline phosphatase (ALP) of 106, international normalized ratio (INR) of 1.9, and lactate dehydrogenase (LDH) of 2269; urinalysis was grossly normal with trace blood and mucus. All workups for underlying causes including autoimmune, infectious, drug-induced, and ischemia were negative. She was started on IV vitamin K and lactulose and transferred to our hospital for evaluation; she was kept under observation with supportive care. Within one week, her liver function tests improved, her INR was back to normal, and she was discharged in a medically stable condition.

**Table 1:** Laboratory values INR: international normalized ratio; LDH: lactate dehydrogenase; PT: prothrombin time

Lab values									
Alanine aminotransferase, U/L	Aspartate aminotransferase, U/L	Total protein, g/dL	Direct bilirubin, mg/dL	Total bilirubin, mg/dL	Alkaline phosphatase, U/L	LDH, U/L	Hemoglobin, g/dL	PT, seconds	INR
2425	2164	5.8	3.0	3.6	106	2269	14.9	25.7	1.90

## Discussion

Artemisinin, also known as qinghaosu in Chinese, is derived from the leaves of the sweet wormwood plant, Artemisia annua [3].^ ^In China, it has been utilized as an herbal remedy for a long time. Although it is a popular ingredient in many herbal supplements produced by US companies, until recently, it was only available in the United States through the Centers for Disease Control and Prevention (CDC) as part of an investigational new drug program [3]. In 2009, the Food and Drug Administration (FDA) approved artemether-lumefantrine, an artemisinin combination therapy, for the treatment of malaria under the brand name Coartem by Novartis [4].^ ^Artemisinin-based treatments are considered safe, effective, and well-tolerated for the treatment of malaria caused by P. falciparum, with few reported side effects [4]. While there have been reports of liver toxicity from ingestion of various herbal preparations, no cases of liver toxicity have been reported specifically due to an artemisinin-containing herbal supplement. However, the FDA's Center for Food Safety and Applied Nutrition has received reports of adverse events related to the use of artemisinin-containing dietary supplement products [3].

In the described case, the symptoms, past medical history, and progression of the patient's condition suggest that her acute liver injury was likely caused by taking an herbal supplement containing artemisinin over a period of three weeks. No other underlying cause of acute liver injury was found. Upon discontinuing the use of the herbal supplement, the patient's symptoms and liver function tests started to show improvement. Further research is needed to establish a direct link between artemisinin and liver damage.

A review of 108 trials involving 9,241 patients found that only 0.9% had elevated AST associated with artemisinin derivatives [5]. The elevated liver enzymes observed in patients treated with artemisinins for malaria are generally attributed to malaria, not the artemisinins. Generally, the recommended oral dose of artesunate is 4 mg/kg/day for three days, in combination with other drugs, for acute malaria. The use of artemisinin herbal supplements in the reported case was substantially longer (three weeks) than the routine treatment for malaria [5].

In a case report published by the CDC, a 52-year-old man presented with fatigue and dark urine; he, similar to our patient, had received a six-week course of an herbal supplement containing artemisinin after seeing a naturopathic physician who had attributed his chronic abdominal discomfort to a parasitic infection [5]. This patient developed symptoms one week into therapy, whereas our patient developed symptoms three weeks into therapy. Lab findings show elevated AST, ALT, total bilirubin, and ALP like our patient. This patient had no other risk factors for acute liver injury, and other etiologies were thoroughly ruled out. His condition improved upon the cessation of the supplement, as was the case with our patient. 

The FDA regulates herbal supplements differently compared to food, over-the-counter drugs, and prescription drugs. Under the Dietary Supplement Health and Education Act of 1994, the manufacturer is accountable for the safety of the supplement, and the FDA takes action against unsafe supplements only after they are on the market [6].^ ^This difference in regulation between supplements and pharmaceuticals raises concerns about quality control, proper usage, and recommended indications. Herbal supplements may also interact with other medications, altering their effects, and may lead to potential toxicity [6]. Healthcare providers should be aware of the possibility that patients may be taking artemisinin-containing supplements and consider asking about their usage during evaluations for hepatitis. Any adverse events linked to the use of artemisinin-containing supplements should be reported to the FDA [6].

## Conclusions

We reported a case of acute liver injury caused by the intake of supplements containing artemisinin derivatives. It is important to discourage the use of these derivatives for malaria prevention or for possible parasitic infections as they have been deemed ineffective by the World Health Organization (WHO) and may cause significant harm. This case also highlights the need for further research to better understand the pathophysiology and the risk of liver injury from herbal dietary supplements.
